# Visual Perception-Based Statistical Modeling of Complex Grain Image for Product Quality Monitoring and Supervision on Assembly Production Line

**DOI:** 10.1371/journal.pone.0146484

**Published:** 2016-03-17

**Authors:** Jinping Liu, Zhaohui Tang, Jin Zhang, Qing Chen, Pengfei Xu, Wenzhong Liu

**Affiliations:** 1 College of Mathematics and Computer Science, Hunan Normal University, Changsha, Hunan, China; 2 Key Laboratory of High Performance Computing and Stochastic Information Processing of Ministry of Education of China, Changsha, Hunan, China; 3 School of Information Science and Engineering, Central South University,Changsha, Hunan, China; 4 School of Automation, Huazhong University of Science and Technology, Wuhan, China; IUMPA—Universitat Politecnica de Valencia, SPAIN

## Abstract

Computer vision as a fast, low-cost, noncontact, and online monitoring technology has been an important tool to inspect product quality, particularly on a large-scale assembly production line. However, the current industrial vision system is far from satisfactory in the intelligent perception of complex grain images, comprising a large number of local homogeneous fragmentations or patches without distinct foreground and background. We attempt to solve this problem based on the statistical modeling of spatial structures of grain images. We present a physical explanation in advance to indicate that the spatial structures of the complex grain images are subject to a representative Weibull distribution according to the theory of *sequential fragmentation*, which is well known in the continued comminution of ore grinding. To delineate the spatial structure of the grain image, we present a method of multiscale and omnidirectional Gaussian derivative filtering. Then, a product quality classifier based on sparse multikernel–least squares support vector machine is proposed to solve the low-confidence classification problem of imbalanced data distribution. The proposed method is applied on the assembly line of a food-processing enterprise to classify (or identify) automatically the production quality of rice. The experiments on the real application case, compared with the commonly used methods, illustrate the validity of our method.

## Introduction

The modern industry gradually advances with worldwide competition and cooperation toward complicated, high-speed, and large-scale development. Product quality [1] is the driving force for every enterprise to stay competitive. In industrial production, product appearance, including the attributes of color, size, surface coarseness, and various defects on the product surface, is an effective visual indicator of internal quality, e.g., ingredient and durability, to a certain extent. Thus, the appearance of a product greatly influences consumer preferences and, consequently, the market value of an enterprise.

In earlier years, product quality was inspected manually through experience and naked-eye observation, which was a time-consuming and labor-intensive task. However, such an inspection method is out of date. An increasing number of manufacturers search for economical and effective ways to monitor and supervise product quality [2]. Effective, low-cost, and on-line inspection technologies on the assembly production line are urgently needed in mass production processes.

In the past decade, considerable efforts have been devoted to online product quality monitoring based on computer-vision systems, given that most types of products can be characterized with corresponding surface visual attributes [3] such as color, object shape and dimension, and surface defects. Thus far, computer-vision systems have been successfully applied in automobile[4], aerospace [5], food processing [6], medical treatment [7,8], textiles [9], electronics [10], nonferrous metallurgy[11,12],and many other industries [13].

In consideration of their functions, the existing computer-vision inspection systems can be generally categorized into two types: quantitative measurement of the physical properties of products and intelligent inspection of product qualities (or working conditions).

In quantitative measurement [14], common image processing techniques, e.g., image enhancement, segmentation, and morphological feature extraction, are usually applied in delineating target objects to measure their physical properties such as size, shape, and color. In these applications, the captured images generally contain clear foreground(target objects) and background regions with distinct contrastive difference. The target objects to be measured in the majority of real applications even have regular geometric shapes. Thus, the commonly used image processing techniques usually exhibit good processing performance in inspecting these target objects.

**Fig 1:**
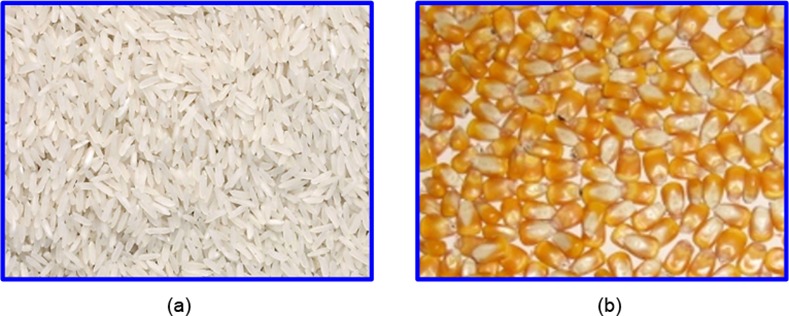
Complex grain images. (a) Rice image, (b) Corn image.

Conversely, in qualitative inspection or production condition perception, the obvious foreground or background cannot be always clearly distinguished from the captured images, such as machine vision-based grain-quality classification [2] and textile-quality inspection [15]. Fig 1 displays two kinds of visual images captured from the assembly lines of rice processing and corn processing. Fig 1 shows that these images are composed of a large number of local homogeneous fragmentations (or particles) without distinct foreground and background. Thus, proposing effective image segmentation methods to delineate and analyze a single target object in images is slightly difficult. The essential information from these visual images for product quality inspection should not be simply obtained from certain local fragmentations or a few particles but should be synthesized from the overall visual appearance reflected by the spatial distribution or the organization of the local fragmentations in the entire observation field. This kind of visual feature for product quality perception, which is widely known as the image texture, is inevitably related to the probabilistic distribution of the image pixels in some special regions [16,17]. Image texture provides significant information for product quality inspection and intelligent perception of the operating conditions in processing monitoring. This important feature exists in nearly all kinds of natural images. However, it cannot be effectively described with a computer.

Researchers have presented various image-texture analysis methods, which can be mainly divided into three categories: simple statistical, structural expression, and model-based methods [18].

Simple statistical methods do not attempt to understand the hierarchical structure of image texture. The most extensive methods among these approaches include gray level co-occurrence matrix (GLCM)[19], gray level run length matrix (GLRM), and local binary pattern(LBP) [20] based texture feature extraction methods, as well as their varieties. These methods cannot achieve a meaningful description of the visual images because these extracted statistics are not related to the human vision system; thus, applying them successfully in product quality monitoring is difficult [21].

Structural expression methods [22] represent image texture through well-defined primitives. Therefore, an image can be described with a hierarchy of spatial arrangements of those predefined primitives. These methods are used primarily for artificial texture analysis, and the complex spatial structures of natural texture images cannot be characterized easily.

Model-based methods attempt to interpret image texture with some special stochastic models, which suppose that image pixels in a local region are equivalent to random sampling points with independent identical distribution. They attempt to describe the relationship between a special image pixel and its adjacent pixels through a certain probability model, such as hidden Markov random field model [23]. Although model-based methods provide a promising idea for the effective description of image texture, current model-based methods conduct limited theoretical analyses of the latent spatial distribution characteristics of these complex grain images. They also do not consider the characteristics of human visual perception adequately. Thus, the visual appearance with respect to the microheterogeneity, spatial stochastic distribution, complexity, and uncertainty exhibited in natural images leads to a great challenge in understanding visual information in a computer-vision inspection system [11].

The human vision system possesses a remarkably intelligent perceptual ability. Shape, size, distance, color, surface roughness, and object motion can be effectively perceived with this intelligent perceptual ability and semantic concepts can be quickly formed for a comprehensive understanding of the observed objects with their environment. Intelligent decision and process automation can be achieved if the visual inspection system can take advantage of the human visual perception characteristics. Although the majority of the mechanisms and functions of the biological vision system are not yet fully understood, many studies have proved that not all of the information of the observed objects can be processed and retained in the biological visual perception process, that is, only a small amount of information is essential to the biological vision system. Studies have shown that the most important information for visual perception in the biological vision system is mainly the information regarding the shapes of objects (or local homogeneous patches or particles) with their spatial layouts, namely, the spatial structure of the objects randomly piled in the vision field.

The statistical modeling of the spatial structure information (texture) plays a key role in the biological vision perception [24]. With respect to visual images, the spatial structures or organizations of local fragmentations are randomly distributed in the microcosmic perspective. However, they exhibit special structural characteristics in a macroscopic perspective, which can be described with some special statistics. The corresponding statistical models[25,26] can also be used as a priori model for image processing. Thus, intensive research on the statistical modeling of the visual image based on human visual perceptual characteristics should be conducted to deduce the corresponding high-level abstract information from a large number of redundant image data for process monitoring.

This study analyzed intensively the statistical distribution characteristics of the spatial structures of complicated grain images with a large number of local homogeneous fragments and particles to extend the perceptive ability of the industrial vision system. These statistical distribution characteristics cannot be described with the traditional image-texture analysis method effectively. The Weibull distribution(WD) process for the spatial organization and distribution of the complex grain images was discussed and proved theoretically in advance based on the theory of *sequential fragmentation*, which is well known in the processes of continued comminution. A novel image filter based on multiscale and omnidirectional Gaussian derivative was presented to characterize the omnidirectional spatial structure appearance under various observation scales. The essential spatial structural appearance of the complex grain images was characterized with the WD model. A kind of product quality classifier based on sparse multikernel–least squares support vector machine (SMK–LSSVM) was established to solve the inaccurate classification and recognition problem of imbalanced data distribution. The proposed method was applied to a food-processing factory for automatic classification and recognition of the rice quality on an assembly line. Extensive comparative experiments were conducted to validate the effectiveness of the proposed method.

## Statistical Modeling of Image Spatial Structures

### WD Process of Grain Image

Given the statistical organization of the texture elements, the essential visual appearance of an image is determined with the spatial distribution of the microscopic structures. For example, an object in the viewing field inevitably fragments the scene into two regions, e.g., an internal region and an external region. The visual scene remains stable until sufficient objects randomly distribute and fragment the scene into a great number of fragmentations or local homogeneous particles [27].

The collected visual images contain abundant local fragmentations or particles if the resolution power of the visual sensors is sufficiently high. Each tiny local fragmentation or particle has a consistent spatial tone. The decrease of the power resolution results in the integration of adjacent local fragment structures; thus, coarse fragmentations or particles are generated in the images. On the contrary, the increase of the power resolution leads to the fragmentations of coarse particles to multiple fine particles. Therefore, given the local fragmentation organization of the images, the distribution of the local fragment particles in the visual image is equivalent to a continued fragmentation process of continued comminution in the ore grinding process. According to the theory of *sequential fragmentation*, the probability distribution of the illumination intensity for a local patch (fragmentation) in an image shows a power-law distribution, which can be described by the following formula[28,29]:

$$f\left(x^{\prime }\to x\right)={\left(\frac{x}{\beta }\right)}^{\lambda -1}$$ (1)

where *x*′→*x* represents the process of decomposing a coarse fragment structure *x*′ into a fine fragment structure *x*; *β* is the average mass (or volume) of the particles in the mill of the ore grinding processes, which can be represented in this study by the average illuminant contrast of the local fragment structures (particles) in the image; and *λ* is a free parameter, which satisfies *λ*≥0. The visual image is composed of multiple local fragments; thus, the pixel contrast statistical distribution of the fragment structures (particles) is the result of an integral over various power-law distributed patches caused by each particle [27].

$$n\left(x\right)=c\int_{x}^{\infty }n\left(x^{\prime }\right)f\left(x^{\prime }\to x\right)dx^{\prime }$$ (2)

where *n*(*x*) represents the histogram distribution of the pixels whose illuminant intensity is between *x* and *x*+*dx*. Thus, *n*(*x*) applies the statistics of all the particles with the contrast of *x*′>*x*. Formula (1) is substituted into Formula (2), and let *c* = 1/*β*. Then, *n*(*x*) can be attained by solving the following equation:

$$n\left(x\right)={\left(\frac{x}{\beta }\right)}^{\lambda -1}\int_{x}^{\infty }n\left(x^{\prime }\right)d\left(\frac{x^{\prime }}{\beta }\right)$$ (3)

By solving Eq 3, the integration over a sufficient number of power laws yields a typical WD [27,29].

$$n\left(x\right)=N_{T}{\left(\frac{x}{\beta }\right)}^{\lambda -1}\cdot e^{-\frac{1}{\lambda }{\left(\frac{x}{\beta }\right)}^{\lambda }}$$ (4)

where $N_{T}=\int_{0}^{\infty }n\left(m\right)dm$ is a normalized parameter.

The resolution power of the visual sensor in the real application cannot be infinite. Thus, the fragmentation process of the local particles in the grain image inevitably ceases, and the particle details always tend to be stable. The statistical distribution of the spatial structure of the grain images merely corresponds to the debris particles with a local contrast larger than *x*. Therefore, the statistical distribution of the spatial structure of the grain image can be described with the WD model of integral form. It is given by

$$N\left(>x\right)=\int_{x}^{\infty }n\left(x'\right)dx^{\prime }=Ce^{-\frac{1}{\lambda }{\left|\frac{x}{\beta }\right|}^{\lambda }}$$ (5)

where $C=1/\int_{-\infty }^{+\infty }e^{-\frac{1}{\lambda }{\left|\frac{x}{\beta }\right|}^{\lambda }}dx=\frac{\lambda }{2\lambda^{\frac{1}{\lambda }}\beta \Gamma \left(\frac{1}{\lambda }\right)}$ is a normalized constant, which is only related to the model parameters *λ* and *β*. Γ(*x*) is a gamma function and $\Gamma \left(x\right)=\int_{0}^{\infty }t^{x-1}e^{-t}dt$.

### Parameter Estimation

The probability density function of the WD model of the integral form is as follows:

$$p\left(x|\lambda ,\beta \right)=\frac{\lambda }{2\lambda^{\frac{1}{\lambda }}\beta \Gamma \left(\frac{1}{\lambda }\right)}e^{-\frac{1}{\lambda }{\left|\frac{x}{\beta }\right|}^{\lambda }}$$ (6)

The most important information regarding this model is the model parameters *λ* and *β*, which can be estimated with the maximum likelihood estimation (MLE) method.

Given that *X* = {*x*_1_,*x*_2_,⋯,*x*_*n*_} is the sampling data, which obeys the integral-form WD model, the corresponding log-likelihood function ln *L*(*X*|*λ*, *β*) indicates how well the model describes the sampling data, which is shown as follows:

$$\text{ln}\;L\left(\lambda ,\beta |X\right)=\text{ln}\;\prod_{i=1}^{n}\frac{\lambda }{2\lambda^{\frac{1}{\lambda }}\beta \Gamma \left(\frac{1}{\lambda }\right)}e^{-\frac{1}{\lambda }{\left|\frac{x_{i}}{\lambda }\right|}^{\lambda }}$$ (7)

The model parameters can be estimated by setting the partial derivative of ln *L*(*X*|*λ*, *β*) over *λ* and *β* to be equal to zero, as shown in Eqs 8 and 9, respectively:

$$\frac{\partial }{\partial \beta }\text{ln}\;L\left(X|\lambda ,\beta \right)=-\frac{n}{\beta }+\frac{1}{\beta }\sum_{i=1}^{n}{\left|\frac{x_{i}}{\beta }\right|}^{\lambda }=0$$ (8)

and

$$\frac{\partial }{\partial \lambda }\text{ln}\;L\left(X|\lambda ,\beta \right)=\frac{1}{\lambda^{2}}\left[\lambda n+n\;\text{ln}\;\lambda -n+n\Phi \left(\frac{1}{\lambda }\right)+\sum_{i=1}^{n}{\left|\frac{x_{i}}{\beta }\right|}^{\lambda }\right]-\frac{1}{\lambda }\sum_{i=1}^{n}\left[{\left|\frac{x_{i}}{\beta }\right|}^{\lambda }\text{ln}\;\left|\frac{x_{i}}{\beta }\right|\right]=0$$ (9)

where $\Phi \left(x\right)=\frac{d}{dx}\text{ln}\;\Gamma \left(x\right)=\frac{\frac{d}{dx}\Gamma \left(x\right)}{\Gamma \left(x\right)}$ is the digamma function.

The parameter *λ* can be calculated by eliminating *β* in Eq 9. Then, the following is obtained:

$$\zeta \left(\lambda |X\right)=1+\frac{\text{ln}\;\lambda }{\lambda }+\frac{1}{\lambda }\Phi \left(\frac{1}{\lambda }\right)-\sum_{i=1}^{n}\frac{{\left|x_{i}\right|}^{\lambda }\left(\text{ln}\;\left|x_{i}\right|-\frac{\text{ln}\sum_{i=1}^{n}{\left|x_{i}\right|}^{\lambda }-\text{ln}\;n}{\lambda }\right)}{\sum_{i=1}^{n}{\left|x_{i}\right|}^{\lambda }}\;=0$$ (10)

Eq 10 does not have a close-form solution. Thus, we can solve it by employing Newton–Raphson method, a gradient-based root-finding method. The main steps are as follows:

Given an initial *λ*_0_, the iterative procedure is repeated as

$$\lambda_{k+1}=\lambda_{k}-\frac{\zeta \left(\lambda_{k}|X\right)}{\frac{\partial }{\partial \lambda_{k}}\zeta \left(\lambda_{k}|X\right)}$$ (11)

where

$$\frac{\partial }{\partial \lambda }\zeta \left(\lambda |X\right)=\frac{1}{\lambda^{2}}-\frac{\text{ln}\;\lambda }{\lambda^{2}}-\frac{\varphi \left(\frac{1}{\lambda }\right)}{\lambda^{3}}-\frac{\Phi \left(\frac{1}{\lambda }\right)}{\lambda^{2}}-\sum_{i=1}^{n}\frac{\left[{\left|x_{i}\right|}^{\lambda }\text{ln}\left|x_{i}\right|\psi \left(X,\lambda \right)\right]\sum_{i=1}^{n}{\left|x_{i}\right|}^{\lambda }-\sum_{i=1}^{n}{\left|x_{i}\right|}^{\lambda }\text{ln}\;\left|x_{i}\right|\left[{\left|x_{i}\right|}^{\lambda }\psi \left(X,\lambda \right)\right]}{{\left(\sum_{i=1}^{n}{\left|x_{i}\right|}^{\lambda }\right)}^{2}}$$ (12)

In Eq 12, $\psi \left(X,\lambda \right)=\text{ln}\left|x_{i}\right|-\frac{\text{ln}\sum_{i=1}^{n}{\left|x_{i}\right|}^{\lambda }-\text{ln}\;n}{\lambda }$ and $\varphi \left(x\right)=\frac{d}{dx}\Phi \left(x\right)$ is the trigamma function.

The iterative procedure is repeated until the estimation *λ*_*n*_ converges, namely, a sufficiently accurate value is reached. After achieving *λ*, *β* can be calculated from Eq 8.

### Perceptual Meaning of WD Model

According to the theory of *sequential fragmentation* [29], the spatial structure of the grain image is generally confined to be subjected to the WD model of the integral form. The integral WD model is an effective physical description for the widely used empirical model, namely, generalized Laplacian or generalized Gaussian model [30]. It is also well applied in image denoising, coding, and image retrieval [24]. The WD model can represent a series of classical statistical distribution shapes by changing the model parameters. For example, when *λ* = 1, WD becomes the exponential distribution with a mean value of *β*. When *λ* = 2, WD becomes Gaussian distribution (GD). For a small value of *λ*, WD is basically close to the symmetric power-law distribution. Some studies have also shown that the shape parameter is directly related to image fractal dimension [29]. The fractal dimension of an image is *D*_*f*_ = −3*λ*.

Studies have shown that the parameters of the WD model can completely characterize the spatial structures (spatial layout or organization of local patches or particles) of a grain image comprised of a great number of local fragmentations. Researchers have determined that the distribution model parameters are directly related to the visual perception characteristic of biological vision systems [27]. With respect to the WD model denoted in Eq 6, *λ* is the shape parameter that reflects the particle size of the visual image texture, and *β* is the scale parameter directly related to the illuminant contrast of the image. The WD model has a direct connection with the human visual perception. We can conduct a correlation analysis of the WD model with the commonly used human perception properties.

The salient perception properties of a texture image, which include coarseness or fineness, regularity, roughness, and directionality, are some of the visual characteristics mentioned in previous studies [31,32].The WD model can effectively explain these human perception properties.

**Fig 2:**
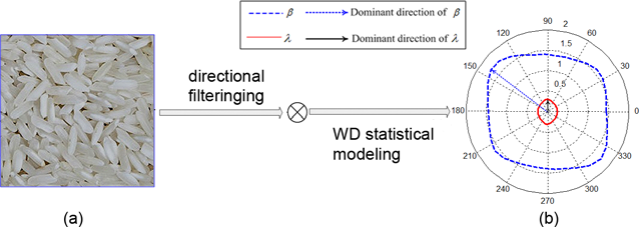
Perception “directionality” of grain image via WDMPs. (a) Original grain image.(b) The polar plot of WDMPs *λ* and *β* of the directional filtering responses of the grain image displayed in (a). The perception “directionality” is reflected by the dominant direction information of parameters *λ* and *β*.

(1) Coarseness or fineness is a fundamental perception attribute when human beings observe and cognize the world (or the projection of the world, e.g., texture images) [31]. In general, the larger the element size is in the grain image, the coarser it is felt for human visual perception, which is innately related to the scale of observation. The perceptual property of “coarseness” can be captured effectively with WD model parameter(WDMP)*λ*. For example, if we zoom in on a grain image, a coarse image appears. The coarseness can be reflected with the shape parameter *λ* of the WD model because the shape parameter *λ* is apparently the reflection of the particle size according to the theory of *sequential fragmentation*. In general, image magnification is accompanied by an increase in resolution power. Then, other details are captured in the field of vision. The details consequently fragment the coarse patches to fine particles or homogeneous regions. According to the theory of *sequential fragmentation*, this process does not affect the distribution shape of the spatial structure, that is, the statistical distribution of the local particles still obeys the WD model. In the extreme case, few particles or even only one particle exists in the field of vision because of the increasing resolution power of the visual sensor together with magnification caused by the decrease of the distance between the lens and the observation field. Then, the WD model slowly converges to a power-law distribution in accordance with Eqs 1, 4 and 5, namely, it effectuates the small value of the shape parameter *λ* of the WD model [27].

(2) Regularity is a perceptual property of the placement rule variations of the texture element (local particles). In general, a fine texture image tends to be perceived as regular. The coarseness or fineness of an image can be indicated by the shape parameter *λ* of the WD model. However, in the extreme case, several particles or even just one particle exists in the receptive field, namely, the image can be distinguished by obvious foreground objects and background regions. Then, the WD model is rejected, and the spatial distribution of the captured scene can be depicted either by power-law distribution or as regular texture. Alternatively, the exhibited statistical distribution shape is often multimodal when the grain image includes numerous fine particles fully filled in the entire receptive field [27]. This phenomenon can be reflected with the estimated shape parameter *λ* of the WD model. The resultant is usually *λ* ≫ 2, which is the overfitting result of the heavy tails of WD distribution. In this case, the spatial distribution of the grain image is perceived to be regular.

(3)Roughness is originally a tactile property of a surface. Humans can perceive it from the visual characteristics of the grain images. In the assessment of the attribute of “roughness,” we initially correct the illumination differences to eliminate the influence of different light conditions. As addressed by Geusebroek [27], the scale parameter *β* is an indicator of the height variations of the texture, and the shape parameter *λ* is the indicator of the granularity of the particles in the grain image. Thus, the perception property “roughness” is reflected by granularity or coarseness, which is measured by the shape parameter *λ*. It is also reflected by the height variation of the texture, which is attained from the scale parameter *β*. Thus, the combination of these two parameters can effectively indicate the roughness of the grain image.

(4)Directionality is a global sense over the entire given region. It indicates the dominant orientation of the texture, which is caused by the shapes of the texture elements as well as their placement rules. The parameters of the WD model do not include the direct shape information of textons (particles). However, the placement of textons (particles) can be implicitly characterized with WDMPs. Studies[27] have demonstrated that the anisotropy of grain size can be described with the dominant direction of the shape parameter *λ*. Anisotropy in texture shadows (or contrast) can be reflected in the dominant orientation on the scale parameter *β* of the WD model. In other words, grain image may exhibit two types of anisotropy. The first type is caused by the particle size, whereas the second type is caused by the contrast variations of particles. Thus, if we fully consider the overall structure information of the grain image, WDMPs can effectively describe the human perception-related “directionality” with the corresponding dominant orientation information. Fig 2 demonstrates the relationship of the perception “directionality” of a grainy image with the WDMPs. Fig 2(A) displays a typical grain image (rice image), whose statistical modeling results obtained with the WD model are plotted in the polar coordinate form, as shown in Fig 2(B). Before statistical modeling, the grain image is filtered with180 directional filters in [0–2π]. Fig 2(B) illustrates that the dominant direction of WDMPs *λ* and *β* conforms to the perception “directionality” of the grain image.

## Image Spatial Layout Characterization

### Gaussian Derivative Filtering (GDF)

The local spatial layout of an image can be completely determined with Taylor expansion of the image at a given point because the image function is the discretization form of a continuous 2D function. A local observing pixel in an image is set as the original observation point labeled as *I*(0, 0).Then, any other pixel (*x*, *y*) in the local spatial structure around the original visual observation point can be determined with Taylor expansion. The approximate expression of the Taylor formula for *I*(*x*,*y*) is

$$\hat{I}\left(x,y\right)=I\left(0,0\right)\left\{\begin{array}{l} 1+\left[xI_{x^{1}y^{0}}+yI_{x^{0}y^{1}}\right]+\frac{1}{2!}\left[x^{2}I_{x^{2}y^{0}}+2xyI_{x^{1}y^{1}}+y^{2}I_{x^{0}y^{2}}\right]+\\ \frac{1}{3!}\left[x^{3}I_{x^{3}y^{0}}+3x^{2}yI_{x^{2}y^{1}}+3xy^{2}I_{x^{1}y^{2}}+yI_{x^{0}y^{3}}\right]+\cdots +\\ \frac{1}{n!}\left[\sum_{i=0}^{n}C_{n}^{i}x^{i}y^{n-i}I_{x^{i}y^{n-i}}\right]+\cdots \end{array}\right\}$$ (13)

The preceding equation indicates that the observed value of a visual pattern in an image is determined with the weighted accumulative addition of the image spatial structures over sufficient spatial observation scales. Differential item $I_{x^{i}y^{n-i}}$ in Eq 13 directly relates to the spatial layout of the image *I*, representing its most important spatial structure information. $I_{x^{i}y^{n-i}}$can be generated with GDF.

$$I_{x^{i}y^{n-i}}\left(x,y\right)=I\left(x,y\right)*G_{x^{i}y^{n-i}}\left(x,y,\sigma \right)$$ (14)

where $G_{x^{i}y^{n-i}}\left(x,y,\sigma \right)$ represents Gaussian derivative filter, whose derivative orders are *i* and *n-i* in *x* and *y* direction respectively; and *i*≥0; *n-i*≥0. σ is the scale parameter of the Gaussian function.

**Fig 3:**
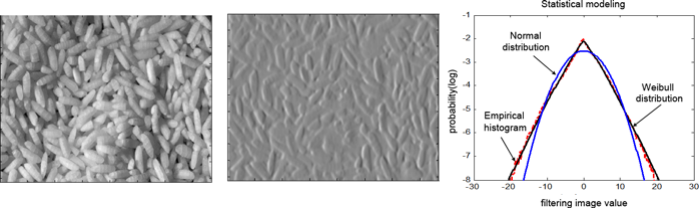
Statistical modeling of image spatial structure. (a) Original rice image *I*.(b) GDF result (*I* * *G*_1,*σ*_) of rice image in (a). (c) Statistical modeling of filtering image with WD and GD models.

We denote *G*_*κ*,*σ*_ as a *k*-order Gaussian derivative filter, where *k* = *i*+(*n-i*), to simplify the description. Fig 3A and 3B show the original rice image and its corresponding spatial structure, respectively, computed with $I*G_{1,\sigma }\left(I_{x^{1}y^{0}}\right)$. Fig 3C displays the statistical modeling results of the filtering image resulting from the WD and Gaussian distribution (GD) models, whose vertical coordinates recording the probability density are the natural logarithm results. The statistical modeling results in Fig 3C explicitly reveal that the WD model is an effective tool to model the distribution shape of the image spatial structure, whose distribution fitting performance is considerably better than that of the GD model.

### Omnidirectional GDF

The use of *G*_*k*_ inimage filtering can reflect the spatial structure information of an original image in *x* and *y* directions, namely, the filtering results only reflect the spatial organization of the local homogeneous patches in the corresponding directions in accordance with *G*_*k*_. However, some spatial structures (e.g., the rice image in Fig 2A) in the visual image have strong directionality. The size and shape of rice differ with rice varieties and rice quality. Thus, the grain structure of the rice image in the assembly line exhibits obvious directional features. A multidirectional filter for omnidirectional feature extraction must be constructed to consider fully the multidirectional spatial structural details of the visual information. Thus, directional filtering information should be introduced into the construction of the traditional Gaussian derivative filter.

If *f*^*θ*^ represents the directional operation of a function *f*, then *G*^*θ*^_*κ*,*σ*_ represents the result of *G*_*κ*,*σ*_ after rotating at an angle of *θ*. According to the research findings of Freeman [33], the image filtering results *G*^*θ*^_*κ*,*σ*_ over any angle *θ* can be computed with the following formula:

$$G^{\theta }{}_{\kappa ,\sigma }\left(x,y\right)=\sum_{i=1}^{M}k_{i}\left(\theta \right)G^{\theta_{i}}{}_{\kappa ,\sigma }\left(x,y\right)$$ (15)

Gaussian derivative filter with any rotational angle can be constructed through the accumulative weighted addition of some special directional Gaussian derivative filters in several limited directions to obtain the spatial structural details of a visual image in any direction. The best way to achieve the optimal expression of *G*^*θ*^_*κ*,*σ*_ is to obtain the minimum number of GDF bases. If the minimum number of GDF bases is *M*, we transform *G*_*κ*,*σ*_ into *G*_*κ*,*σ*_(*r*, *ϕ*) under the polar coordinates, where $r=\sqrt{x^{2}+y^{2}}$, *ϕ* = *angle*(*x*, *y*). Then, we can obtain the following result through Fourier series decomposition:

$$Fourier\left(G_{\kappa ,\sigma }\left(r,\phi \right)\right)=\sum_{n=0}^{N}a_{n}\left(r\right)e^{in\phi }$$ (16)

Eqs 15 and 16 reveal that the optimal number *M* is equivalent to the number of nonzero harmonic components in the Fourier series of *G*_*κ*,*σ*_(*r*, *ϕ*). In other words, *M*is equal to the sum of nonzero number in *a*_*n*_(*r*). Therefore, *k*_*i*_(*θ*) is the solution to the following equation by solving the Fourier transformation under the polar coordinates:

$$\left(\begin{array}{c} 1\\ e^{i\theta }\\ \cdots \\ e^{iN\theta } \end{array}\right)=\left(\begin{array}{cccc} 1 & 1 & \cdots & 1\\ e^{i\theta_{1}} & e^{i\theta_{2}} & \cdots & e^{i\theta_{M}}\\ \vdots & \vdots & \ddots & \vdots \\ e^{iN\theta_{1}} & e^{iN\theta_{2}} & \cdots & e^{iN\theta_{M}} \end{array}\right)\left(\begin{array}{c} k_{1}\left(\theta \right)\\ k_{2}\left(\theta \right)\\ \vdots \\ k_{j}\left(\theta \right) \end{array}\right)$$ (17)

*k*_*i*_(*θ*) can be obtained with Eq 17 by selecting some specific GDF bases. For example, the following results can be obtained:

$$G^{\theta }{}_{1,\sigma }\left(x,y\right)=\text{cos}\left(\theta \right)G_{x^{1}y^{0}}\left(x,y\right)+\text{sin}\left(\theta \right)G_{x^{0}y^{1}}\left(x,y\right)$$ (18)

$$G^{\theta }{}_{2,\sigma }={\text{cos}}^{2}\left(\theta \right)G_{x^{2}y^{0}}\left(x,y\right)+2\;\text{sin}\left(\theta \right)\text{cos}\left(\theta \right)G_{x^{1}y^{1}}\left(x,y\right)+{\text{sin}}^{2}\left(\theta \right)G_{x^{0}y^{2}}\left(x,y\right)$$ (19)

**Fig 4:**
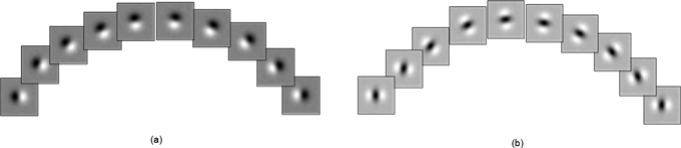
Gaussian derivative filters with specific directions. **(a)** *G*^*θ*^_1,*σ*_**.(b)** *G*^*θ*^_2,*σ*_.

Fig 4A and 4B show the directional Gaussian derivative filters of *G*^*θ*^_1,*σ*_ and *G*^*θ*^_2,*σ*_, respectively, in [0∼180°]. The image spatial structure characteristics under any direction can be obtained by constructing the omnidirectional Gaussian derivative filters (ODGDFs)through the statistical modeling of the filter responses with the WD model.

### Comparison with Gabor filters

The proposed ODGDFs are slightly similar to the commonly used Gabor wavelets(GWs). Both GWs and the proposed ODGDFs act in the same way as band pass filters. However, essential differences exist in the choice of the frequency domain.

**Fig 5:**
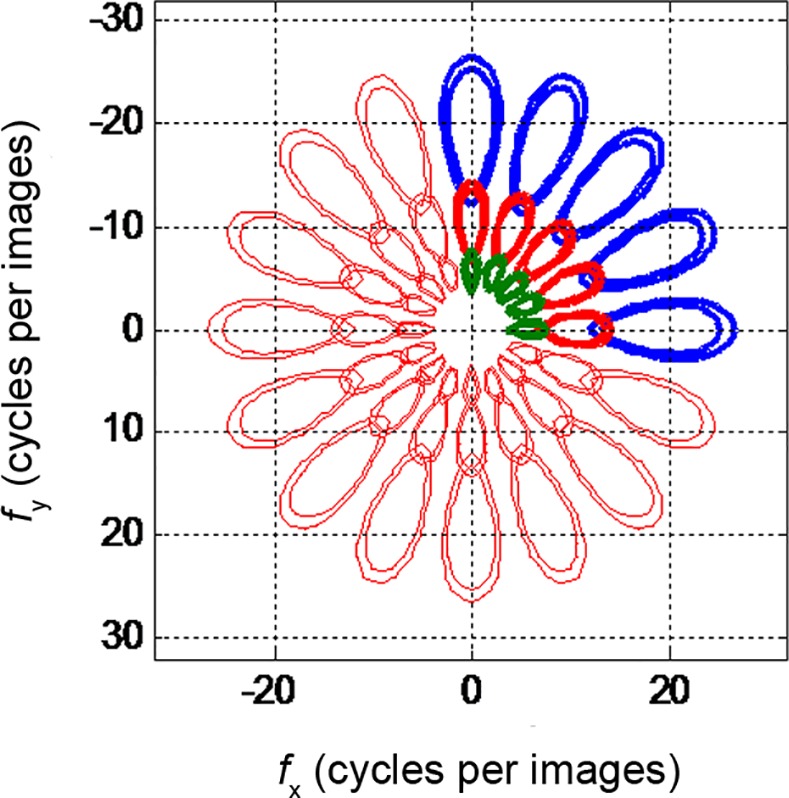
Plot of frequency responses of Gabor filters under different scales and different orientations.

Gabor transform is a special case of short-time Fourier transform. Images can be decomposed into *M* channels of different orientations and scales through Gabor wavelet transform. The Fourier transform of a Gabor filter is the convolution of the Fourier transform of the harmonic function and the Fourier transform of the Gaussian function. Fig 5 plots the half-value of the Gabor filters in the frequency plane tuned to different frequencies and orientations.

The proposed ODGDFs are based on the property of the Taylor expansion of the 2D function. As stated earlier in this paper, if we set a local observing pixel in an image as the original observation point labeled as *I*(0, 0), then any other pixel (*x*, *y*) in the local spatial structure around the original visual observation point can be determined with Taylor expansion.

$$\hat{I}\left(x,y\right)=I\left(0,0\right)\sum_{k=0}^{\infty }\frac{1}{k!}\sum_{i=0}^{k}C_{k}^{i}x^{i}y^{k-i}I_{x^{i}y^{k-i}}$$ (20)

**Fig 6:**
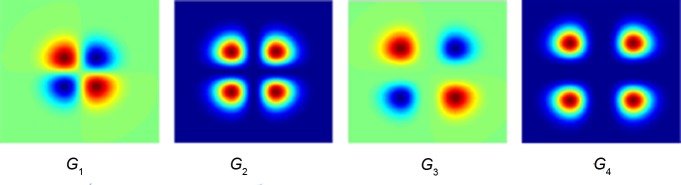
Frequency responses of *G*_*κ*_.

Eq 20 indicates that the observed value of a visual pattern is gained by integrating the image spatial structures over sufficient spatial observation scales. The differential item $I_{x^{i}y^{n-i}}$ in Eq 20 directly relates to the spatial structure of image *I*, representing the most important spatial organization information of the local homogeneous particles or patches in the grain images. $I_{x^{i}y^{n-i}}$ can be generated through GDF. Multiscale and multidirectional Gaussian derivative filters are introduced in this study to obtain the image spatial structure features of diverse scales and orientations. The difference of Gabor filters with the proposed Gaussian derivative filters can be observed from the frequency responses. Fig 6 displays the frequency responses of *G*_*κ*_ under different orders of *κ*, where *σ* = 1 and the direction is *θ* = 0°. In contrast to the plot in Fig 5, the proposed ODGDFs evidently capture the different information of the processed image in the frequency domain against the commonly used GWs.

## Application Case

### Overview of Machine Vision-based Rice Quality Inspection System

Today, people pay close attention to the quality of their food. Different consumer groups have different concerns about food quality. For example, some people attach considerable importance to the color and luster of food, whereas others pay significant attention to the shape or ingredients. For a food-processing enterprise, automatic classification and recognition of the product quality play a key role in the food production process.

Rice is a major source of dietary energy and protein with regard to human nutrition and caloric intake. It is the most widely consumed staple food in many countries, particularly China, which has the largest population in the world. Thus, intelligent and automatic rice processing quality grading or classification, as well as packaging, have represented the core competitiveness of each rice processing company. The majority of rice processing enterprises have employed automatic classification technology instead of inefficient and subjective manual inspection of rice processing quality to provide high-quality products (e.g., rice) and to reduce processing cost. In recent years, automatic monitoring of rice quality based on machine vision has drawn extensive attention locally and internationally.

Brosnan [2,6] reviewed the developments of machine vision-based quality inspection technology of food products in various applications in the food industry in the early years. A detailed summary of the existing computer vision-related external quality inspection of fruits and vegetables can be found in the literature [34],which presents the principles, developments, and applications of these systems. This study also determined that a machine vision-based external quality inspection and grading system is important and necessary in the postharvest preprocessing stage and that it has become a common and scientific tool in industrial and agricultural manufacturing automation.

The existing literature indicates that people tend to be concerned about the physical properties of each individual rice grain in earlier years, such as surface gloss, physical shape, size, and other characteristics of each rice grain[35]. As addressed in the surveys[2,36], the first step of these methods is rice image segmentation, which is the foundation of gaining proper descriptions of surface color, shape, and size of each individual rice grain. After image feature extraction, a kind of classifier, such as neural network(NN), support vector machines(SVM) or other supervised pattern recognition methods, is established to achieve the automatic grading or classification of rice processing quality[37].

Many experiments verified in earlier years the effectiveness of the aforementioned methods, which can achieve a high grading accuracy (higher than 90% recognition); however, in the practical application, many problems remain, such as particle image segmentation accuracy and image processing efficiency (the highest record is only 1,200particles per minute[36]).

In recent years, researchers have been expected to bypass the time-consuming and unsatisfactory image segmentation process in rice image processing. Therefore, researchers tend to focus on the spatial variations (structural distribution) in the intensity or color space of the rice images. In particular, they paid significant attention to the spatial structure features of rice image, usually called image texture[38].

Jackman[39] determined that further analysis with image texture in addition to the traditional image features of the rice image is expected to achieve a sophisticated description to obtain high classification accuracies. Researchers previously focused on the texture feature of a rice image for rice processing quality inspection. The commonly used texture description methods are some simple second statistics based on GLCM,GLRM, and the histogram description of the pixel difference, as well as other mathematical description methods[39].

**Fig 7:**
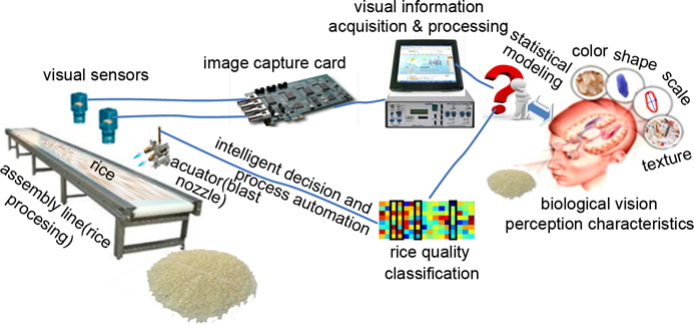
Machine vision-based rice quality monitoring system.

The proposed method is applied to a food-processing enterprise in South China to verify the effectiveness of this method by identifying the grain quality automatically. Fig 7 displays the schematic of the visual monitoring system. In the assembly line, several conveyors are equipped to parallel process the rice, among which each the conveyor belt is approximately 95mm wide. The rice is evenly distributed on the conveyor belt for automatic classification and reprocessing. The capacity of each conveyor belt reaches 45 kg/h. The visual sensors are equipped above the conveyor belt perpendicular to the belt surface for rice quality monitoring. In the experiments, an IL-P3-2048 Linear CCD is mounted, whose pixel dimensions are14 *μ*m × 14 *μ*m, and the active pixel per line is 2,048 with 20MHz data rate per tap. The F24mm/2.8 fixed-focus lens is used, and the pixel resolution of each frame of rice image for quality inspection is 2048 × 128. The aforementioned WD process of the image spatial structure indicates that the WD model is the innate distribution of the image statistics with a large number of local fragmentations, which do not vary with the resolution power of the visual sensor or the size of the images. Other resolutions of the rice images achieve the analogous classification performance.

The rice image is processed and analyzed through the method described in the second section to realize the online identification of rice quality on the assembly line. When the rice quality is inferior, the actuator (a blast nozzle) is automatically controlled to blast air. Then, the low-quality rice is blown away from the conveyor belt for rice reprocessing to classify the rice of different quality and to yield high-quality rice for consumers.

### Extraction of Spatial Structure Features of Rice Images

**Fig 8:**
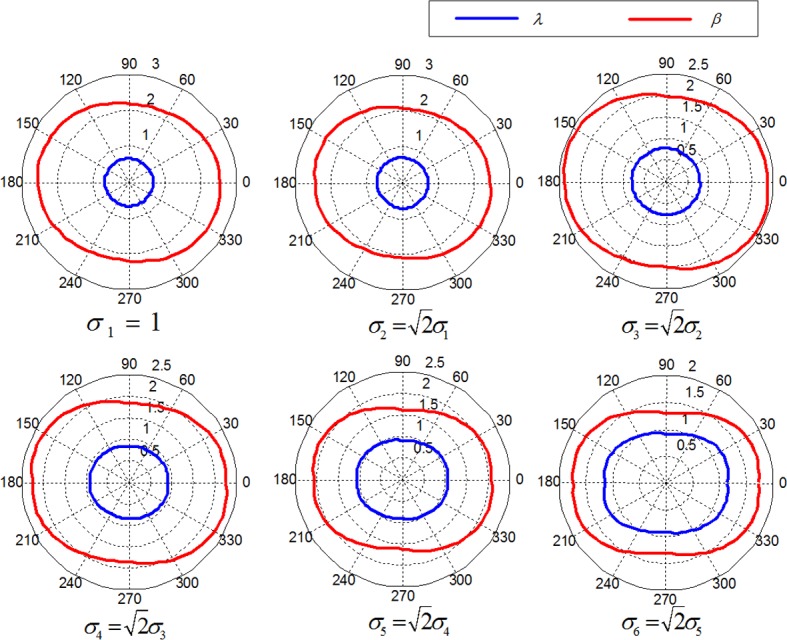
Polar diagrams of WDMPs of omnidirectional spatial structures.

*N*-direction ODGDFs are used for image spatial organization characterization to consider fully the multidirectional structural feature. Every disparity between the two adjacent directions is 360°/*N*. In terms of each filtering response by ODGDF, the WDMPs of the rice image under a special observation scale are obtained. Several scales *σ* are used in this study to obtain multiscale image spatial structure details. Fig 8 shows the polar diagram of the WDMPs of the omnidirectional spatial structure of a rice image in Fig 3A under different observation scales.

If the spatial observation scale for image spatial structure analysis is *σ*, then, for any image, the statistical distribution feature *f*_*img*,*σ*_ of the omnidirectional spatial organization under observation scale *σ* can be expressed as

$$f_{img,\sigma }=\left[\beta_{\theta_{1},\sigma }\beta_{\theta_{2},\sigma },\cdots ,\beta_{\theta_{N},\sigma },\lambda_{\theta_{1},\sigma },\lambda_{\theta_{2},\sigma },\cdots ,\lambda_{\theta_{N},\sigma }\right]$$ (21)

where $\beta_{\theta_{i},\sigma }$ and $\lambda_{\theta_{i},\sigma }$ represent the WDMPs^*β*^ and *λ*, respectively, under the scale *σ* in the direction of *θ*_*i*_. If *T* dimensions of Gaussian observation scales [*σ*_1_,*σ*_2_,⋯,*σ*_*T*_] exist, then the statistical distribution features of the multiscale omnidirectional image spatial structure can be characterized as follows:

$$f_{img}=\left[f_{img,\sigma_{1}},f_{img,\sigma_{2}},\cdots ,f_{img,\sigma_{T}}\right]$$ (22)

### Construction of Rice Processing Quality Classifier

Intelligent product quality inspection is a pattern classification or recognition problem. In the statistical learning and classification methods, support vector machine(SVM)based on the s*tructural risk minimization* principle is the most recent nonlinear classifier, which is a promising tool to successfully resolve the small size issues in pattern recognition [40]. The major drawback of the primal SVM in classification is the high computational burden for the constrained optimization programming. LSSVM is proposed to solve the computational problem by solving linear equations instead of a quadratic programming problem [41]. However, the sparseness property of SVM disappears because of the choice of the L2-norm in the LS–SVM case. In reality, the sparseness property of a classifier is most important because it allows for fast and accurate evaluation of new data points [42].

A method of sequential minimal optimization was introduced into the pruning process for sparse LSSVM to impose sparseness in LSSVM solutions [43]. However, the pruning method is a time-consuming process because of the iterative computing steps for the removal of samples, which do not directly affect the support values of other samples. Thus, a method of achieving the sparse solution of the LSSVM classifier, namely, SMK–LSSVM classifier, is adopted in the present study. In this method, the kernel matrices are reduced with Schmidt orthogonalization theory to reduce computational complexity.

### Least Squares Support Vector Machine (LSSVM) Classifier

With respect to the classifier, the spatial structural feature *x*_*t*_ of any rice image *t* is used as the input vector of the LSSVM classification model, the output *y*_*t*_ represents the corresponding rice quality labeled based on manual assay and naked-eye observation results. For practical application, this study only considers two types of rice quality, which are defined as

$$y_{t}=\left\{ \begin{array}{ll} -1 & "\text{high quality}"\;\text{rice}\\ +1 & \;\text{other quality rice} \end{array}\right.$$ (23)

Given a number of *N* training samples ${\left\{x_{t},y_{t}\right\}}_{t=1}^{N}$, the LSSVM classification model function *f*(*x*_*t*_ → *y*_*t*_) can be defined as follows:

$$y_{t}\left[w^{T}\Phi \left(x_{t}\right)+b\right]=1-\xi_{t}$$ (24)

where *w* is the weighting vector, *b*is the deviation vector, *ξ*_*t*_ is the potential classification error, and Φ(*x*) is a nonlinear mapping function through which the input data are mapped to the high-dimensional data space. The corresponding optimization function can be constructed as follows:

$$\begin{array}{l} \text{min}\;J\left(w,b,\xi \right)=\frac{1}{2}w^{T}w+\frac{r}{2}\sum_{t=1}^{N}\xi_{t}^{\;2}\\ s.t.\left\{ \begin{array}{l} y_{t}\left[w^{T}\Phi \left(x_{t}\right)+b\right]=1-\xi_{t}\\ \xi_{t}\geq 0 \end{array}\right. \end{array}$$ (25)

The rice quality classification is a two-category classification problem, *y*_*t*_^2^ = (±1)^2^ = 1. Thus, $\sum_{t=1}^{N}\xi_{t}^{\;2}=\sum_{t=1}^{N}{\left(y_{t}\xi_{t}\right)}^{2}=\sum_{t=1}^{N}{\left\{y_{i}-\left[w^{T}\Phi \left(x_{t}\right)+b\right]\right\}}^{2}=\sum_{t=1}^{N}e_{t}^{\;2}$, where *e*_*t*_ = *y*_*t*_ − (*w*^*T*^Φ(*x*_*t*_) + *b*).

The corresponding Lagrange function can be constructed as follows to solve the aforementioned optimization problem:

$$L\left(w,b,e,a\right)=J\left(w,b,\xi \right)-\sum_{t=1}^{N}a_{t}\left\{\left[w^{T}\Phi \left(x_{t}\right)+b\right]+e_{t}-y_{t}\right\}=\frac{1}{2}w^{t}w+\frac{r}{2}\sum_{t=1}^{N}e_{t}{}^{2}-\sum_{t=1}^{N}a_{t}{}^{2}\left\{\left[w^{T}\Phi \left(x_{t}\right)+b\right]+e_{t}-y_{t}\right\}$$ (26)

where *a*_*t*_ is the Lagrange multiplier. The partial differential equations of variables under Karush–Kuhn–Tucker optimality condition can be obtained as follows:

$$\left\{ \begin{array}{l} \frac{\partial L}{\partial w}=0\Rightarrow w=\sum_{t=1}^{N}a_{t}\Phi \left(x_{t}\right)\\ \frac{\partial L}{\partial b}=0\Rightarrow \sum_{t=1}^{N}a_{t}=0\\ \frac{\partial L}{\partial e_{t}}=0\Rightarrow a_{t}=re_{t}\\ \frac{\partial L}{\partial a_{t}}=0\Rightarrow y_{t}=w^{T}\Phi \left(x_{t}\right)+b+e_{t} \end{array}\right.$$ (27)

*w* and *e* are eliminated, and the solution of the optimization problem in Eq 25 can be transformed to the following linear equations:

$$\left[\begin{array}{cccc} 0 & 1 & \cdots & 1\\ 1 & K\left(x_{1},x_{1}\right)+r^{-1} & \cdots & K\left(x_{1},x_{1}\right)\\ \vdots & \vdots & \ddots & \vdots \\ 1 & K\left(x_{n},x_{1}\right) & \cdots & K\left(x_{n},x_{n}\right)+r^{-1} \end{array}\right]\left[\begin{array}{c} b\\ a_{1}\\ \vdots \\ a_{n} \end{array}\right]=\left[\begin{array}{c} 0\\ y_{1}\\ \vdots \\ y_{n} \end{array}\right]$$ (28)

where *K*(*x*_*i*_, *x*_*j*_) = Φ(*x*_*i*_)Φ(*x*_*j*_) is the kernel function, which satisfies Mercer’s condition. After computing *a* and *b* with Eq 28, the classifier parameter *w*can be achieved through further calculation by substituting *a* and *b* into Eq 27. Consequently, the LSSVM-based automatic rice quality classifier can be obtained as follows:

$$f\left(x\right)=sgn\left(\sum_{t=1}^{N}a_{t}K\left(x,x_{t}\right)+b\right)$$ (29)

### SMK–LSSVM Classifier

In the real application, the data distribution is frequently imbalanced, for example, the number of samples regarding “high quality” is larger than the number of samples about “other quality” rice. In the generalized LSSVM classification model described in Eq 25, each sample is set at a constant penalty parameter *r*, which is not a highly effective solution for the classification of the imbalanced distribution data. Thus, different penalty factors are provided for each sample to treat the samples differently in accordance with their importance. Thus, the optimization problem in Eq 25 is slightly modified as follows:

$$\text{min}\;J\left(w,b,\xi \right)=\frac{1}{2}w^{T}w+\frac{r}{2}\sum_{t=1}^{N}c_{t}\xi_{t}^{\;2}$$ (30)

where *rc*_*t*_ is the penalty factor that corresponds to the training sample *t*. For the computing performance, the samples of the same types are set to the same values as follows:

$$c_{t}=\{\begin{array}{cc} s^{+} & \;y_{t}\in +1\\ s^{-} & \;y_{t}\in -1 \end{array}$$ (31)

Eq 29 shows that the choice of the different kernel function *K*(•,•) greatly affects the classifier performance. Given that the kernel function is the central role of the classifier, a good choice of the kernel is imperative to the success of the kernel-based recognition methods. The radial basis function (RBF) kernel has good local learning ability, whereas the polynomial kernel has excellent global generalization ability [42]. A multikernel-based LSSVM classifier is introduced in this study to achieve a good classification performance by taking advantage of the merits of the RBF and polynomial kernels. Thus, in Eqs 28 and 29, the kernel function *K*(•,•) is constructed as follows:

$$K\left(x,x_{i}\right)=\eta K_{1}\left(x,x_{i}\right)+\left(1-\eta \right)K_{2}\left(x,x_{i}\right)$$ (32)

where *K*_1_(*x*, *x*_*i*_) = (1+*x*_*i*_^*T*^*x*/*c*)^*d*^ and *K*_2_(*x*, *x*_*i*_) = exp(−‖*x* − *x*_*i*_‖^2^ / 2*σ*^2^) represent the polynomial kernel and RBF kernel function, respectively. Schmidt orthogonalization method can be used to build a sparse multikernel LSSVM by reducing the computation.

If a vector [Φ(*x*_1_),⋯,Φ(*x*_*n*_)]^*T*^ represents the mapping expressions of the training samples in the high-dimensional space, each Φ(*x*_*i*_) can be formulated with the combination of column vectors of a transformational matrix [42].

$$\left[\begin{array}{c} \Phi \left(x_{1}\right)\\ \vdots \\ \Phi \left(x_{n}\right) \end{array}\right]=\left[\begin{array}{ccc} \alpha_{11} & \cdots & \alpha_{1n}\\ \vdots & \ddots & \vdots \\ \alpha_{n1} & \cdots & \alpha_{n1} \end{array}\right]\left[\begin{array}{c} \tilde{\Phi }\left(x_{1}\right)\\ \vdots \\ \tilde{\Phi }\left(x_{n}\right) \end{array}\right]$$ (33)

where ${\left[\tilde{\Phi }\left(x_{1}\right),\cdots ,\tilde{\Phi }\left(x_{n}\right)\right]}^{T}$ is a basis vector of the mapped expression, which can realize the sparseness of the multikernel LS–SVM through orthogonalization processing.

According to the Schmidt orthogonalization method, the orthogonalized Φ(*x*_*i*_) can be computed as follows:

$$\Phi_{t+1}\left(x_{i}\right)=\Phi_{t}\left(x_{i}\right)-\left(\Phi_{t}{}^{T}\left(x_{i}\right)v_{t}\right)v_{t}$$ (34)

where $v_{t}=\frac{\Phi_{t}\left(x_{i}\right)}{\sqrt{\Phi_{t}{}^{T}\left(x_{i}\right)\Phi_{t}\left(x_{i}\right)}}$ and Φ(*x*_*i*_)(*i* ∈ [1,⋯,*n*]) are the selected vectors.

Gram’s form of the kernel matrix can be expressed as follows:

$$G\left(a,b\right)=\Phi {\left(x_{a}\right)}^{T}\Phi \left(x_{b}\right)=K\left(x_{a},x_{b}\right)$$ (35)

Thus,

$$G_{t+1}\left(a,b\right)=G_{t}\left(a,b\right)-\frac{G_{t}\left(a,x_{i}\right)G_{t}\left(a,x_{i}\right)}{G_{t}\left(x_{i},x_{i}\right)}$$ (36)

In the matrix orthogonalization processing, the column vector in column *x*_*i*_ that corresponds to the maximum *G*(*i*, *i*) is selected first. Then, the remaining vectors are orthogonalized as the quasi method. Overall, the rank of the matrix is *γ*. Thus, the sparse kernel matrix can be constructed as follows:

Algorithm 1

________________________________________________________________________________

Step 1: ${\tilde{G}}_{0}\left(p,p\right)=K\left(x_{p},x_{p}\right)$ is set.

Step 2: The kernel matrix is initialized.

            For *t* = 0: *γ*−1

$${\tilde{G}}_{0}\left(t,p\right)=K\left(x_{t},x_{p}\right),\;t=t+1$$

            End

Step 3: For *t* = 0: *γ*−1

            *x*_*i*_ is selected corresponding to the maximum *G*(*i*, *i*) and *ind*(*t*) = *i* is set.

            For *s* = *t*+1: *γ*−1

$${\tilde{G}}_{t+1}\left(s,p\right)={\tilde{G}}_{t}\left(s,p\right)-\frac{{\tilde{G}}_{t}\left(s,p\right){\tilde{G}}_{t}\left(ind\left(t\right),ind\left(s\right)\right)}{G_{t}\left(ind\left(t\right),ind\left(t\right)\right)}$$

            End

$${\tilde{G}}_{t+1}\left(p,p\right)={\tilde{G}}_{t}\left(s,p\right)-\frac{{\tilde{G}}_{t}\left(t,p\right)G_{t}\left(t,p\right)}{G_{t}\left(ind\left(t\right),ind\left(t\right)\right)}$$

            End

________________________________________________________________________________

The optimal model parameters *c*^+^ and *c*^−^, the polynomial kernel function parameters *d*, and the RBF kernel function parameter σ can be determined with particle swarm optimization (PSO) algorithm to improve the recognition performance of the rice quality classification model:

$$\begin{array}{l} v_{i,d}\left(t+1\right)=dv_{i,d}\left(t\right)+c_{1}r_{1}\left[P_{i,d}-x_{i,d}(t)\right]+c_{2}r_{2}\left[P_{g,d}-x_{i,d}\left(t\right)\right]\\ x_{i,d}\left(t+1\right)=x_{i,d}\left(t\right)+v_{i,d}\left(t+1\right) \end{array}$$ (37)

where *x*_*i*,*d*_, *v*_*i*,*d*_, *P*_*i*,*d*_, and *P*_*g*,*d*_ represent the current position, current speed, current optimal position of the particle, and optimal position of the particle, respectively. *d* is a constant in[0,1], which is referred to as the internal coefficient;*c*_1_ and *c*_2_ are the learningrates;*r*_1_ and *r*_2_are the stochastic coefficients in [0,1].

### Rice Quality Classification Results

Five varieties of rice image samples are collected from a rice-processing assembly line. The corresponding rice qualities are manually calibrated based on the measurement of the nutrition component combination from the voting results of several experts by observing the rice appearance. The five rice varieties are supposedly marked as *ω*_1_ ∼ *ω*_5_.

The total sample numbers of the five rice varieties are 800, 756, 789, 824, and 802. In the classification experiments, if $N_{T_{i}}$ samples of the rice variety *ω*_*i*_ are selected as the test samples and the remainder are used for classifier training, then the classification error of the test samples of rice variety *ω*_*i*_ can be calculated with the following equation:

$$CE_{i}=\frac{1}{N_{T}}\sum_{t=1}^{N_{T}}\frac{\left|{\hat{y}}_{t}-y_{t}\right|}{2}*100\%$$ (38)

where *y*_*t*_ represents the actual rice quality label, and ${\hat{y}}_{t}$ represents the automatic recognition result of the rice quality. Four experiments are conducted to validate the classification performance of the proposed method. The experiments are as follows:

**Experiment 1:**Verification test. We achieve the rice processing quality classification results with the proposed method, namely, the rice processing quality is classified by the WDMPs of the images with the SMK–LSSVM classifier.

In this experiment, five Gaussian function scales are selected for the sake of extracting the omnidirectional spatial statistics of the rice image under various observation scales, where $\left[\sigma_{1},\sigma_{2},\cdots ,\sigma_{5}]=[0.5,\sqrt{2}/2,1,\sqrt{2},2\right]$. A total of180 directions in [0 ∼ 360°] are applied in this study to construct the Gaussian derivative filter banks for the omnidirectional spatial structure feature analysis. No larger than three-order Gaussian derivative filters (*G*_1_ ∼ *G*_3_) with their combinations are considered. In constructing the classifier (SMK–LSSVM), the regularization factor *r* = 8.4, and the multikernel weighting parameter *η* in Eq 32 is set as 0.4.The polynomial kernel function parameter *d* = 0.25, and the corresponding penalty factors *c*^+^ and *c*^−^ and the RBF kernel function parameter *σ* are optimized with the PSO algorithm. Five repeating experiments are performed. In each independent experiment, the same number of samples (500 samples) is randomly selected for the classifier test and the remaining samples for classifier training. The average classification accuracies of the rice quality by the proposed image feature extraction method with SMK–LSSVM classifier are shown in Table 1.

**Table 1:** Rice quality classification results by WDMP features with SMK–LSSVM classifier.

Gaussian derivative filters	Average classification accuracy of five independent experiments (1−*CE*_*i*_)*100%					
	*ω*_1_	*ω*_2_	*ω*_3_	*ω*_4_	*ω*_5_	Average
*G*_1_	89.67	91.40	89.96	92.90	92.71	91.33
*G*_2_	85.33	89.84	91.34	86.11	89.07	88.34
*G*_1_+*G*_2_	93.33	97.66	91.69	98.45	92.72	94.78
*G*_3_	77.33	82.03	84.42	82.71	82.45	81.79
*G*_2_+*G*_3_	89.00	93.33	88.23	92.28	90.06	90.58
*G*_1_+*G*_2_+*G*_3_	97.33	97.66	97.58	98.77	99.33	98.13

The classification results in Table 1 shows that when only one kind of Gaussian derivative filter is independently chosen for the image space structure feature extraction, the best classification result can be achieved by selecting the first-order Gaussian derivative filter *G*_1_, whose average classification accuracy can reach 91.33%.The rice quality classification performance is relatively poor when only the *G*_3_ filter is used. However, the accurate classification rate can also be slightly higher than80%. This finding demonstrates that the combination of more than one filter significantly improves the classifier performance. Third-order and less than the third-order Gaussian derivative filters can effectively characterize the spatial structural appearance, and satisfactory classification results can be achieved. The experimental results shown in Table 1 reveal that the accurate classification rate of the rice quality of any rice variety is more than 97% when all of the Gaussian derivative filters (*G*_1_+*G*_2_+*G*_3_) are integrated, and the average classification accuracy of the five rice varieties reaches as high as 98.13%, which can effectively meet the imperative demand of the rice-processing factory.

**Experiment 2:** Comparative experiment. The rice quality classification results by different image feature extraction methods integrated with SMK–LSSVM classifier are compared.

The results of some rice quality classification methods based on commonly used machine vision-based technologies are also compared with the aforementioned results in this experiment to evaluate further the performance of the proposed WDMP features. These methods include the image feature extraction methods based on image GLCM and GLRM mentioned in the literature[44], the method based on wavelet analysis (WTA) [45,46], and the commonly used multi-channel texture classification method, namely, Gabor wavelets [21]. The detailed rice image surface texture feature extractions based on the GLCM/GLRM, WTA, and Gabor wavelets are described in Table 2. Five independent repeating experiments are performed in experiment 2, similar to experiment 1. In each experiment, 500 samples of each rice variety are randomly selected for the classifier performance test, and the rest of the samples are used for classifier training. The average rice quality classification accuracy is displayed in Table 3.

**Table 2:** Texture feature extraction based on GLCM/GLRM, Gabor wavelets, and WTA.

Texture features	Features extraction details
GLCM/GLRM	(1) The original image is quantified into images with 8, 32, and 64 gray levels. (2)For each quantized gray-level image, 16 GLCM/GLRM matrices with the displacement of *l*_*i*_ ∈ [2,5,10,15] and the orientation of *θ*_*j*_ ∈ [0°,45°,90°,135°] are computed. (3) Fourteen statistics[47],including energy, moment of inertia, partial correlation, entropy, and coarseness, are extracted to every GLCM/GLRM matrix.(4) The average values of the 14 statistics are computed as the final image texture features for rice quality classification experiment.
Gabor wavelet	(1) The intensity image is used for feature extraction. (2) Forty Gabor filters with five scales and eight orientations are applied. (3) The statistical mean and standard deviation of the amplitude response of the Gabor filtering image are extracted as the image feature descriptor.
WTA	(1) Three color spaces, namely, HIS, CIE, and L*a*b*,are used for the image analysis.(2)Db4 wavelet is used for multiscale decomposition in each independent color space until the image size under the largest scale is no less than 8 × 8. (3) A total of 15 characteristics [45](e.g., energy and color covariance) are calculated based on the image wavelet detail coefficients under each decomposition scale to constitute the image feature vector.

**Table 3:** Rice quality classification results by GLCM/GLRM and WTA features with SMK–LSSVM classifier.

Image feature selection	Average classification accuracy of five independent experiments (1−*CE*_*i*_)*100%					
	*ω*_1_	*ω*_2_	*ω*_3_	*ω*_4_	*ω*_5_	Average
WTA	75.00	75.80	80.40	71.40	77.40	76.00
GLCM	77.20	75.20	72.40	75.80	75.00	75.12
GLRM	77.20	78.40	76.20	79.00	80.40	78.24
GLCM+GLRM	79.56	80.20	75.00	78.80	84.60	79.63
Gabor wavelet	84.80	86.34	82.00	81.40	77.80	82.46

The classification performance is significantly lower than the proposed WDMP features presented in this study. The average classification rates of the five rice varieties are all lower than 85%. The average accuracies of the WTA, GLCM, and GLRM methods are poor at less than 80%. The combination of GLCM and GLRM features can improve the classification performance over some rice varieties. However, the classifier performance cannot be improved obviously on all of the five rice varieties, and the average classification rate remains low(only 79.63%). The test results obtained by the traditional image feature extraction methods with SMK–LSSVM classifier reveal that the methods based on the commonly used GLCM/GLRM, WTA, and Gabor wavelets are not highly suitable for rice image analysis and feature extraction. These methods cannot effectively characterize the most important visual cue of a rice image and the spatial structural appearance of the surface texture structure. Moreover, the extracted statistics by GLCM/GLRM or WTA are not related to the human vision perception, which lacks specific physical significance. Thus, satisfactory rice quality classification performance cannot be achieved with the image features as the input of the proposed SMK–LSSVM classifier by the traditional image analysis methods (GLCM/GLRM or WTA), and the imperative demand of the automatic rice classification, processing, and packaging of the assembly line in the food-processing factory cannot be met.

**Experiment 3:** Comparative experiment. The rice quality classification results by different image feature extraction methods integrated with the commonly used LSSVM classifier are compared.

In this experiment, we mainly test the rice quality classification performance by the proposed rice image statistics, WDMP features, and the traditional image analysis methods (GLCM/GLRM, Gabor wavelets, and WTA) with the widely used LSSVM-based classifier. The experiment operations are the same as those in experiment 1. The rice processing quality classification results by the proposed image analysis method and the traditional methods are reported in Tables 4 and 5, respectively.

**Table 4:** Rice quality classification results by WDMP features with LSSVM classifier.

Gaussian derivative filters	Average classification accuracy of five independent experiments (1−*CE*_*i*_)*100%					
	*ω*_1_	*ω*_2_	*ω*_3_	*ω*_4_	*ω*_5_	Average
*G*_1_	86.34	90.40	89.96	92.90	92.71	90.46
*G*_2_	83.38	89.84	91.34	86.11	89.07	87.95
*G*_1_+*G*_2_	89.68	92.00	90.45	93.45	92.63	91.64
*G*_3_	80.26	84.03	85.42	82.71	82.45	82.97
*G*_2_+*G*_3_	87.45	89.46	91.89	87.94	90.06	89.36
*G*_1_+*G*_2_+*G*_3_	95.34	94.28	95.48	95.27	92.43	94.56

**Table 5:** Rice quality classification results by GLCM/GLRM and WTA features with LSSVM classifier.

Image feature selection	Average classification accuracy of five independent experiments (1−*CE*_*i*_)*100%					
	*ω*_1_	*ω*_2_	*ω*_3_	*ω*_4_	*ω*_5_	Average
WTA	74.64	76.80	78.56	73.48	73.26	75.34
GLCM	76.48	73.20	74.68	74.68	75.12	74.83
GLRM	74.48	75.40	73.86	76.58	80.89	76.24
GLCM+GLRM	78.86	81.20	75.00	76.89	82.24	78.84
Gabor wavelets	82.40	81.80	82.80	80.00	78.80	81.16

By combining the rice quality classification results of experiment 1 with those of experiment 2, we can draw the following conclusions: (1) With regard to the rice quality classification performance, the proposed SMK–LSSVM classifier is better than the traditionally used LSSVM classifier. We can achieve an average classification accuracy as high as 98.13% through the proposed SMK–LSSVM classifier with the proposed ODGDF-based spatial structure statistics of the rice image (*G*_1_+*G*_2_+*G*_3_). However, we can only obtain the classification accuracy at a rate of 94.56% by LSSVM classifier with the same image statistical features. (2) When the traditional image analysis methods (GLCM/GLRM, Gabor wavelets, or WTA) are combined with the LSSVM classifier, the rice quality classification rates of all the five rice varieties are also low, which are obviously inferior to the proposed spatial structure feature extraction method in this study for the effective rice quality classification.

**Experiment 4:** Comparative experiment. The rice quality classification results by different image feature extraction methods integrated with learning vector quantization-neural network(LVQ–NN) classifier are compared.

In addition to the SVM method, NN can model various complex nonlinear systems. It has been also extremely widespread in various pattern recognitions. Among the different architectures of NN, LVQ–NN is the nearest-neighbor pattern classifier based on competitive learning with simple architecture and good pattern classification performance. In this experiment, a LVQ–NN classifier is constructed to test the rice quality classification performance with different image feature extraction methods. The input and output of LVQ–NN are the same as those of the LSSVM method in the previous experiments. The number of hidden layer nodes in the LVQ–NN classifier is set through cross validation, and the number corresponding to the best classification performance is recorded as the number of the LVQ–NN hidden layer nodes. The ultimate number of the hidden layer nodes is set to 24, which can ensure the optimal rice quality classification accuracy. The corresponding classifier training algorithm is LVQ2[48]. The average classification accuracy rates by the LVQ–NN classifier with the proposed spatial structural statistical modeling analysis and the traditional image analysis methods are presented in Tables 6 and 7, respectively.

**Table 6:** Rice quality classification results by WDMP features with LVQ–NN classifier.

Gaussian derivative filters	Average classification accuracy of five independent experiments (1−*CE*_*i*_)*100%					
	*ω*_1_	*ω*_2_	*ω*_3_	*ω*_4_	*ω*_5_	Average
*G*_1_	83.46	86.32	88.96	90.24	90.25	87.84
*G*_2_	84.68	83.24	85.38	87.81	89.27	86.07
*G*_1_+*G*_2_	88.56	90.36	91.36	91.06	89.89	90.24
*G*_3_	78.24	80.45	80.38	83.34	83.25	81.13
*G*_2_+*G*_3_	86.40	92.00	86.68	90.36	90.26	89.14
*G*_1_+*G*_2_+*G*_3_	88.42	90.28	90.48	94.54	92.76	91.29

**Table 7:** Rice quality classification results by GLCM/GLRM and WTA features with LVQ–NN classifier.

Image feature selection	Average classification accuracy of five independent experiments (1−*CE*_*i*_)*100%					
	*ω*_1_	*ω*_2_	*ω*_3_	*ω*_4_	*ω*_5_	Average
WTA	73.24	71.40	78.68	74.45	73.26	74.02
GLCM	74.46	75.00	71.56	74.84	75.00	74.17
GLRM	72.60	75.40	74.56	77.58	82.24	76.47
GLCM+GLRM	77.40	78.20	76.12	79.98	83.68	79.08
Gabor wavelet	82.20	84.20	86.00	80.20	77.80	82.08

The experimental results reveal that the SMK–LSSVM classifier is considerably better than the LSSVM classifier and LVQ–NN classifier, regardless of whether the proposed spatial structure statistics features of the grain image or the traditional image analysis methods are used in the rice quality classification. In addition, the spatial structural features, WDMPs, proposed in this study are highly similar to the human visual perceptual characteristics to a certain extent, which can effectively describe the spatial organization appearance of the complex texture images with a large number of local fragment structures. The proposed method can achieve a considerably high classification accuracy rate with a small number of training samples by combining the classification results in Tables 1 and 7. The proposed method can be effectively applied to automatic rice quality inspection in the real assembly line to realize the automatic classification of grain quality, which provides a means of effective automatic monitoring for the processing and production of high-quality rice.

## Conclusions

This study has analyzed and proved theoretically the WD process of the spatial structure of the complicated texture images by introducing the *sequential fragmentation* theory. A method of omnidirectional and multiscale GDF methods is presented to characterize the most important visual hint of the complex grain images. Based on the statistical modeling of the image spatial structure with the WD model, some statistical distribution shape-related variables with significant visual perceptive meaning are extracted, thereby effectively describing the spatial structure distribution characteristics of the complex textural images. Finally, a product quality classifier-based SMK–LSSVM is constructed, and Schmidt orthogonalization method is introduced to reduce the model computation.

The proposed image analysis method and product classifier are applied to a food-processing enterprise for automatic rice quality inspection on the assembly line to achieve satisfactory classification performance. Exhaustive comparative experiments demonstrate that the proposed method outperforms the traditional image analysis method with the commonly used LSSVM and LVQ–NN classifiers. The proposed method can achieve the accurate classification results of various varieties of rice quality, which lays a foundation for the effective processing and automatic packaging production of high-quality rice.

## Supporting Information

S1 Fig Grain image I for image statistical modeling by WD model. (PPTX)

S1 Table Image statistical modeling resultsof Grain image I by WD model. (XLSX)
