# Supplementary figures and images for: Visual Perception-Based Statistical Modeling of Complex Grain Image for Product Quality Monitoring and Supervision on Assembly Production Line
Source: PLoS One. 2016 Mar 17;11(3):e0146484. doi: 10.1371/journal.pone.0146484 (PMC4795607; doi:10.1371/journal.pone.0146484)

## Slide 1
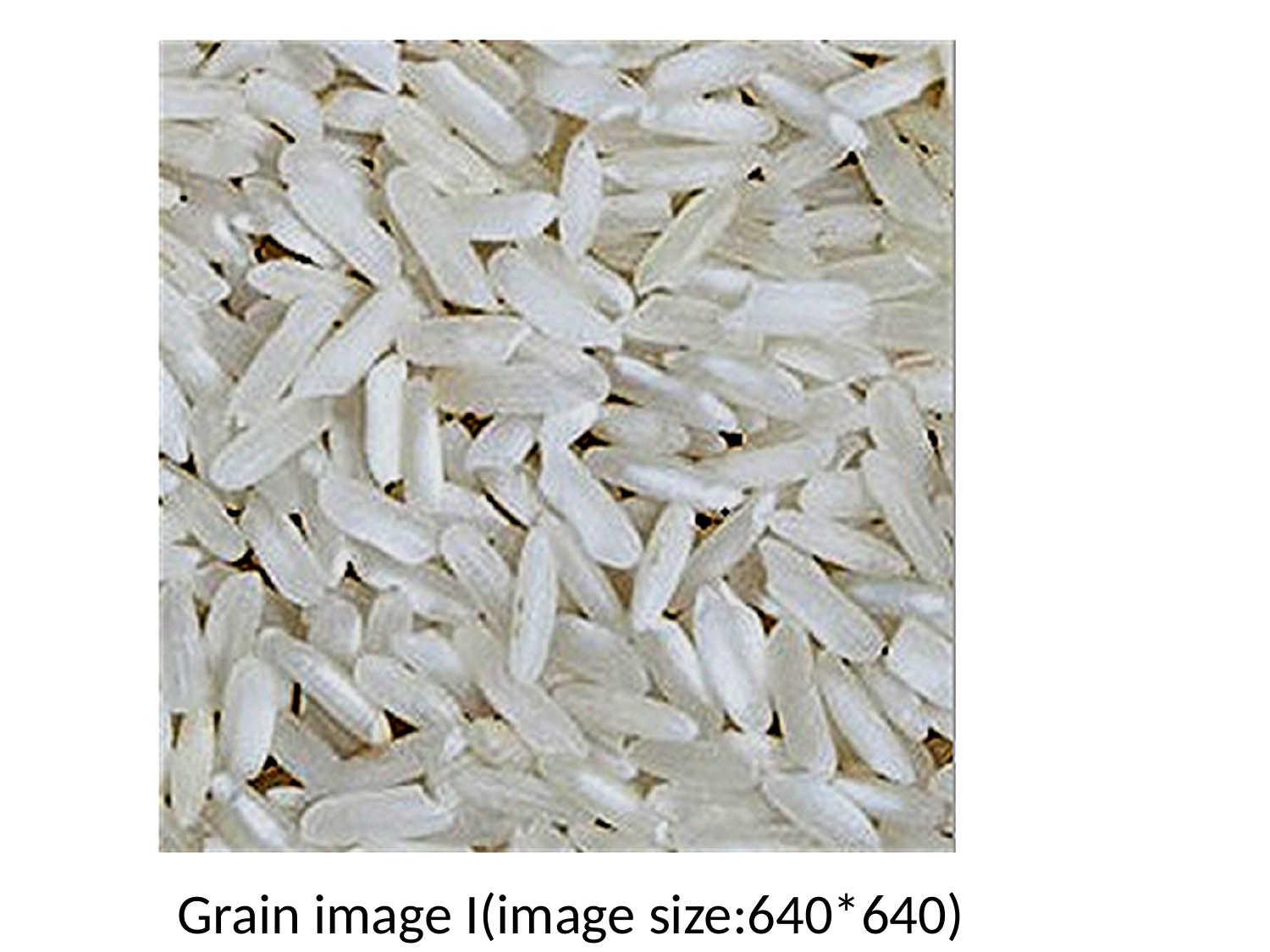

Grain image I(image size:640*640)

Supplement: S1 Fig — (PPTX) [file pone.0146484.s001.pptx]
